# Array Formatting of the Heat-Transfer Method (HTM) for the Detection of Small Organic Molecules by Molecularly Imprinted Polymers

**DOI:** 10.3390/s140611016

**Published:** 2014-06-20

**Authors:** Gideon Wackers, Thijs Vandenryt, Peter Cornelis, Evelien Kellens, Ronald Thoelen, Ward De Ceuninck, Patricia Losada-Pérez, Bart van Grinsven, Marloes Peeters, Patrick Wagner

**Affiliations:** 1 Institute for Materials Research, Hasselt University, Wetenschapspark 1, B-3590 Diepenbeek, Belgium; E-Mails: gideon.wackers@student.uhasselt.be (G.W.); thijs.vandenryt@uhasselt.be (T.V.); peter.cornelis@student.uhasselt.be (P.C.); evelien.kellens@uhasselt.be (E.K.); ronald.thoelen@uhasselt.be (R.T.); patricia.losadaperez@uhasselt.be (P.L.-P.); bart.vangrinsven@maastrichtuniversity.nl (B.G.); marloes.peeters@uhasselt.be (M.P.); patrick.wagner@uhasselt.be (P.W.); 2 IMEC vzw—Division IMOMEC, Wetenschapspark 1, B-3590 Diepenbeek, Belgium; 3 Maastricht Science Programme, Maastricht University, 6200 MD Maastricht, The Netherlands

**Keywords:** heat-transfer method (HTM), molecularly imprinted polymers (MIPs), serotonin, histamine, l-nicotine, array format

## Abstract

In this work we present the first steps towards a molecularly imprinted polymer (MIP)-based biomimetic sensor array for the detection of small organic molecules via the heat-transfer method (HTM). HTM relies on the change in thermal resistance upon binding of the target molecule to the MIP-type receptor. A flow-through sensor cell was developed, which is segmented into four quadrants with a volume of 2.5 μL each, allowing four measurements to be done simultaneously on a single substrate. Verification measurements were conducted, in which all quadrants received a uniform treatment and all four channels exhibited a similar response. Subsequently, measurements were performed in quadrants, which were functionalized with different MIP particles. Each of these quadrants was exposed to the same buffer solution, spiked with different molecules, according to the MIP under analysis. With the flow cell design we could discriminate between similar small organic molecules and observed no significant cross-selectivity. Therefore, the MIP array sensor platform with HTM as a readout technique, has the potential to become a low-cost analysis tool for bioanalytical applications.

## 1. Introduction

**Figure 1.**
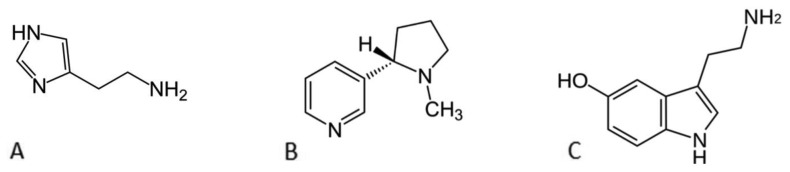
Chemical structure of: (**A**) histamine; (**B**) serotonin and (**C**) l-nicotine.

For different application areas including separation science and purification [1], environmental testing [2,3], biosensors [4,5], drug delivery and diagnostics [6,7], it is of interest to develop chemical sensors that can be tailored for specific analytes. Traditionally, the approach is to design individual sensing elements for certain targets which is a laborious and resource intensive process. The use of molecularly imprinted polymers (MIPs) can overcome these drawbacks [8,9]. MIPs are synthetic receptors that are prepared by copolymerizing functional monomers with crosslinker monomers in the presence of a particular template molecule [10]. After removal of this target, cavities are obtained with a high affinity and selectivity for the corresponding template molecule [11]. Imprinting is a versatile and inexpensive method [12] to obtain recognition surfaces with different selectivity patterns. The MIP can be rapidly tailored to a specific analyte by selecting monomers via computational modeling [13]. It is also versatile since it can be used for the detection of ions [14] and small organic molecules such as neurotransmitters [15] to larger biomolecules including proteins [16] and cells [17]. For biomedical analyses, DNA microarray chips are a successful example for high-throughput parallel processing [18], but MIP microarray systems are still in their developing stages and literature reports are sparse. The first sensor array was reported by Shimizu *et al.* in 2004 [19]. They assembled eight polymers, seven molecularly imprinted and one non-imprinted polymer serving as reference, to successfully differentiate seven biogenic amines with a colorimetric binding assay. Takeuchi *et al.* [20] used UV-vis spectroscopy to monitor the change in analyte concentration. While they managed to extend the method from small organic molecules to proteins, also high levels of cross-reactivity were observed. Qiu *et al.* employed a chemiluminescence sensor array to determine benzenediol isomers with a graphene-magnetite-molecularly imprinted polymer [21]. The usage of a fluorescent marker complicates the readout technique due to the need of a confocal microscope and expensive markers. Only recently, an electrochemical strategy has been developed by Hawari *et al.* [22] who combined a MIP layer with an interdigitated electrode (IDE) as sensor. Upon exposing the sensor platform to different mango volatiles, a shift in the capacitance was observed. This specific response could be correlated to the stage of mango ripeness of the sample. The drawbacks of both optical and electrochemical techniques are that they require expensive readout equipment and analysis is often non-straightforward. In this research an array format will be demonstrated with proof-of-principle experiments performed on the target molecules histamine, serotonin and l-nicotine (Figure 1).

A novel read-out technique has been proposed to detect small organic molecules and cells with MIP type receptors [23,24]. This heat-transfer method (HTM) eliminates the need of sophisticated equipment since it requires only two thermocouples, a proportional-integral-derivative (PID) controller, an adjustable heat source, and ensures fast detection of a variety of target molecules in buffer solutions. This method is very cost-effective, both in terms of the measurement setup and in sample preparation. While with the sensor platform physiologically relevant concentrations could be determined, it was necessary to add a large sample volume and only one sample could be measured at a time [15,17]. In this research we will present an optimized flow cell design which can measure four samples simultaneously, *i.e.*, the quadrants of the flow cell can be functionalized with different MIPs, thereby significantly diminishing the total measurement time. Furthermore, the sample volume is reduced to 1 μL which is a great benefit for analyzing biological samples. Histamine and serotonin are neurotransmitters, which are indispensable for the efficient functioning of a variety of physiological processes [25]. They are similar in chemical structure since they both contain an imidazole ring and an amine functionality. However, their size is different and therefore as a third template molecule l-nicotine was added. l-Nicotine, the major addictive substance in tobacco which is carcinogenic [26], has the same size as histamine and thereby both chemical and physical properties are taken into account when determining the selectivity. We will demonstrate that, upon exposure of the sample to small organic molecules in buffer solutions, the MIPs will only show a response to the target it is designed for. This low cross selectivity, in combination with the miniaturization and the reduction of measurement time, are important steps into implementing the sensor setup for diagnostic applications. Typical label-free MIP biosensors appear as single channel detection platforms. Therefore, they are only capable of sensing one analyte at a time and truly differential measurements are difficult to achieve. When HTM is used as the method of detection, the inter-sample variability often proves difficult to control. However, when a single substrate can be divided into different areas of detection, the variability decreases considerably. Moreover, the quantities of imprinted polymers and target molecules decreases while the speed of consecutive measurements increased by a fourfold, when compared to previous iterations of a HTM setup [5,17,24].

## 2. Experimental Section

### 2.1. Materials

The monomers used for the synthesis of the MIP- and NIP-particles, ethylene glycol dimethacrylate (EGDM), methacrylic acid (MAA), acrylic acid (AA) and acrylamide (AM), were purchased from Acros (Geel, Belgium). For EGDM, MAA and AA the stabilizers were removed by filtration over alumina powder prior to polymerization. The solvent dimethylsulfoxide (DMSO), serving as a porogen was obtained from Acros and the azobisisobutyronitrile (AIBN) initiator was acquired from Fluka (Buchs, Switzerland). The template molecules histamine and serotonin were obtained from Alfa Aesar (Karlsruhe, Germany). l-Nicotine was purchased from Acros. All solvents were of analytical grade.

### 2.2. MIP Synthesis

The synthesis of the MIP particles was performed according to the following recipe. A mixture of the functional monomers, crosslinker monomers and initiator were dissolved in the porogen together with the template molecule. After degassing the solution, polymerization was initiated either by UV-light or by heat. After polymerization, the compound was ground and the template was removed by Soxhlet extraction. The non-imprinted polymer (NIP) was prepared according to the same recipe except for the addition of the target molecule. Therefore, the NIP can be used as a negative control. For full details on the synthesis procedure, see references [15,27,28].

### 2.3. Sensor Chip Preparation

Aluminum substrates (1 × 1 cm, thickness of 1 mm) were polished with sandpaper of increasing grit sizes up to P4000 to achieve a mirror finish. After the polishing and cleaning with acetone, H_2_O and isopropanol the substrates were spincoated (3000 rpm, 500 m/s^2^) with OC_1_C_10_-PolyPhenyleneVinylene (MDMO-PPV). MDMO-PPV, synthesized according to reference [29], serves as an adhesive layer for the MIP- and NIP particles. A polydimethylsiloxane (PDMS) stamp was pressed into the corresponding MIP- or NIP-powder and onto the adhesive layer in order to transfer the particles onto the substrate. By varying the stamp size it is possible to either cover the entire surface of the substrate with a MIP targeted to a certain template or to create four areas coated with MIPs targeted to different templates. Errors in the area-selective deposition of the MIPs can be avoided by covering parts that should not be stamped and using adequately sized stamps. The substrates were placed on a hotplate to heat them to 120 °C (*i.e.*, above the glass transition temperature of MDMO-PPV), which allows the particles to partially sink into the softened PPV layer. After cooling down the samples they were rinsed with isopropanol in order to remove particles that are not fixated onto the substrate. The verification of the deposition and particle loading was performed with an Axiovert 40 inverted optical microscope (Carl Zeiss, Oberkochen, Germany).

### 2.4. Design of Sensor Setup

**Figure 2.**
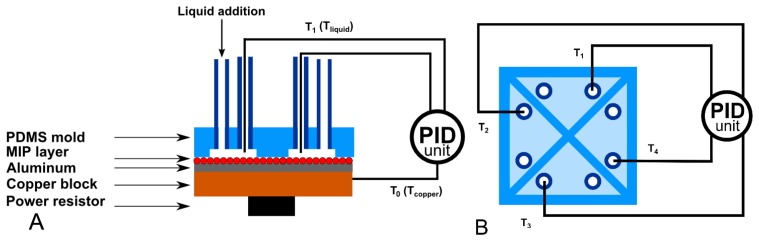
Schematic side view (**A**) and top view (**B**) of the PDMS flow cell and the four thermocouples. The temperature of the copper, T_0_, is strictly controlled. The temperature of the liquid (T_1,2,3,4_) in each quadrant is solely monitored.

The functionalized substrates were placed on a copper block onto which a power resistor (22 Ω, MPH20S, Farnell, Grace-Hollogne, Belgium) was attached. The PDMS flow cell, containing four quadrants with a surface area of 5 mm^2^ and an inner height of 0.5 mm, is placed on top of the sample. The outer dimensions of this stamp are 10 mm wide, 10 mm long and 4 mm high. The volume of each quadrant is 2.5 μL, excluding the volume in the tubing. Two Perspex plates are used to apply a small amount of pressure to the setup which ensures a watertight seal. A miniature thermocouple (type K, diameter 500 μm, TC Direct, Nederweert, The Netherlands) was inserted into the copper block. The temperature (T_0_) of the copper block is controlled at 37.00 ± 0.02 °C to mimic the human body temperature with a PID (P = 1, I = 8, D = 0.1) controller that was designed in-house. A thermocouple is inserted into each of the cavities to monitor the temperature (T_1,2,3,4_) in the liquid at equal height (500 μm) above the sample. The liquid temperature, as monitored by the four remaining thermocouples, is logged by the software once per second. Figure 2 shows a schematic design of the sensor setup. No inlet or outlet is shared and the four quadrants are completely isolated from each other, in order to avoid interference between the four quadrants during a measurement. The developed flow cell can be reused for multiple measurements provided that the setup is cleaned in between with isopropanol when detecting hydrophilic molecules.

To start a measurement, the setup was filled with phosphate buffered saline (PBS) solution (pH = 7.4). The setup was allowed to heat up to 37.00 ± 0.02 °C and left to stabilize for at least one hour. After stabilization the template molecules (histamine, serotonin and L-nicotine) with a concentration of 1 mM in PBS (pH = 7.4) were added, resulting in changes in temperature of the fluid. After calculating the power necessary to keep the setup at a constant temperature this value can then be used to obtain the thermal resistance (R_th_) for each quadrant. R_th_ is defined as $R_{\text{th}}=\frac{T_{\text{o}}-T_{\text{i}}}{P}$, in which P is the power in Watt (W), T_0_ is the temperature in degrees Celsius (°C) of the heat sink (copper block), and T_i_ with *i* = 1,2,3,4 is the temperature of the fluid in each quadrant in degrees Celsius (°C).

## 3. Results and Discussion

**Figure 3.**
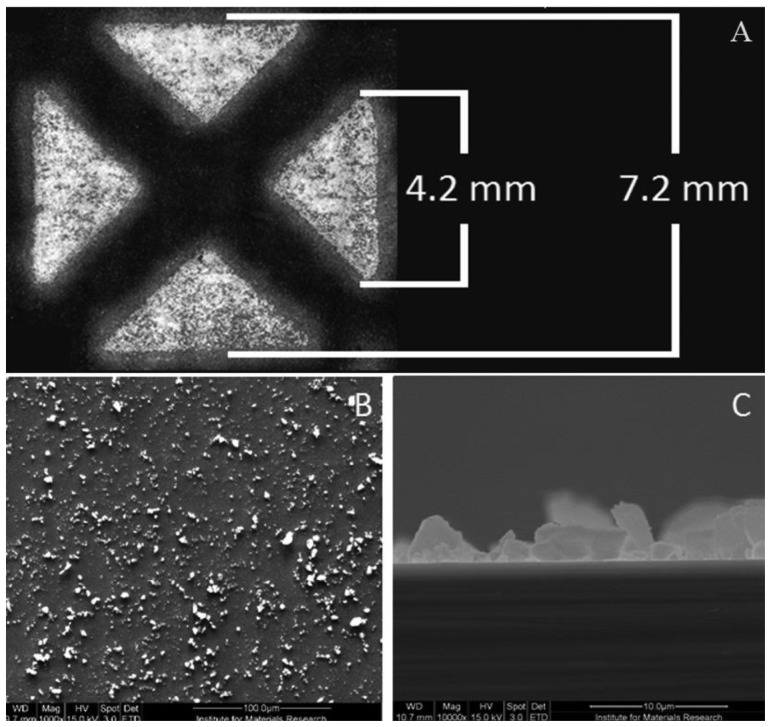
(**A**) The MIPs are distributed over four quadrants with a length of 4.2 mm. Between the quadrants, the interspacing was 2 mm. The total size corresponds to 7.2 mm. The bright spots correspond to areas where the MIP particles cover the glass slide. The optical image was obtained with an Axiovert microscope of Carl Zeiss. (**B**) shows a Scanning Electron Microscopy (SEM) image which was zoomed into a quadrant containing one type of particles. (**C**) demonstrates a side view of the sample.

To determine whether we could functionalize the four quadrants of the setup separately, the MIP particles were stamped onto a glass slide and analyzed by optical microscopy. These substrates were not used for sensor measurements. As can be observed from Figure 3A, the quadrants are well defined, indicating that particles are only deposited on the intended positions. An analogous procedure was performed with the actual aluminum substrates (MIP and NIP functionalized), of which the electron microscope image is displayed in Figure 3B,C. The picture shows the microscope image of the quadrants. The sample has a size of 10 by 10 mm and a thickness of 1 mm.

**Figure 4.**
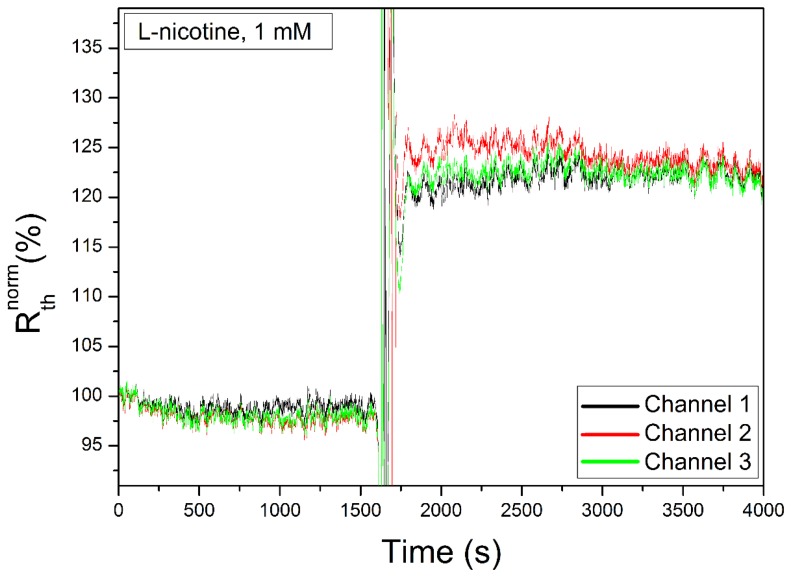
Shows the result of a measurement with three MIP channels, imprinted for l-nicotine. After a stabilization period in PBS buffer, a solution of 1 mM nicotine target in PBS buffer is flushed sequentially through each quadrant of the sample. The overshoot seen at 1600 s is caused by the temporary temperature difference when introducing the test solution originally at room temperature.

There is a clear cross shaped area onto which no MIP particles have bound, indicating that there is a perfect separation between the quadrants. Experiments have been performed to assess the consistency of the data for all quadrants. First, three quadrants, loaded with MIPs for nicotine detection, have been created, together with one reference channel loaded with NIPs. These channels were stabilized in PBS buffer (pH = 7.4). Then, 1 mL solution of 1 mM target molecule has been added sequentially to the four quadrants. The data obtained for the three MIP-stamped channels is shown in Figure 4. These demonstrate a low intra-sample variability.

**Figure 5.**
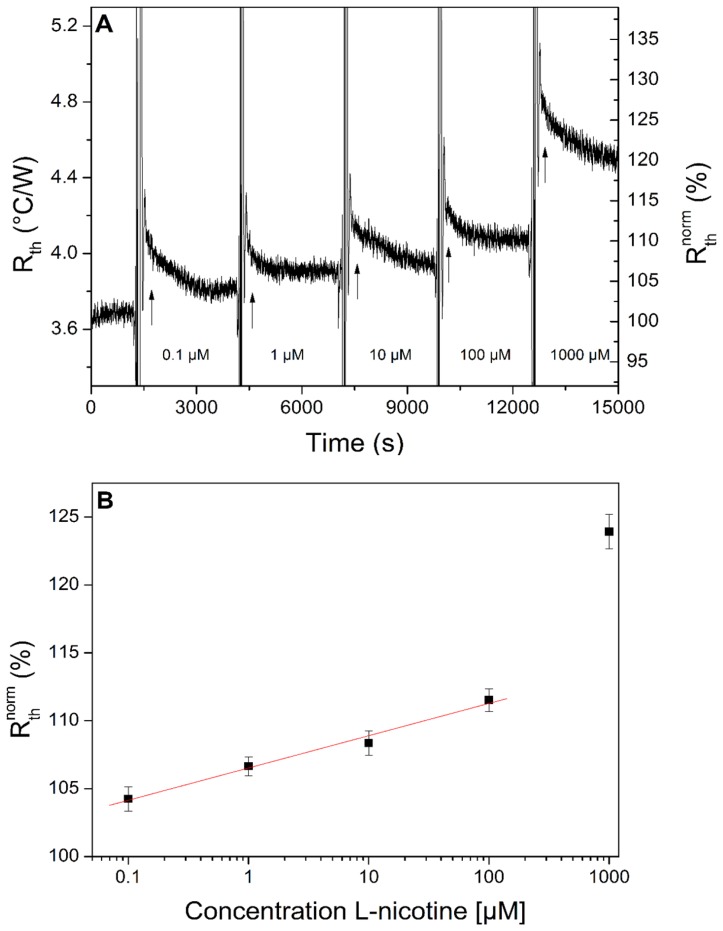
(**A**) shows the raw, unfiltered R_th_ data for five solutions with an increasing L-nicotine concentration on a sensor chip covered with MIPs for l-nicotine detection. The right vertical axis displays the normalized data, expressed as a percentage of the initial R_th_ in PBS. (**B**) is the dose-response curve in which the concentration *versus* the relative response in R_th_ is plotted. The data are not corrected for non-specific adsorption effects.

After verification of the clear separation of the quadrants and their reproducible response characteristics, the performance in terms of the dose-response characteristics for a specific target molecule was determined. Therefore, the setup was functionalized with particles for l-nicotine and analyzed for its response to this target. A substrate onto which l-nicotine MIPs were attached was mounted in the setup and left to stabilize for an hour. Five solutions of l-nicotine in PBS with concentrations of 100 nM, 1 μM, 10 μM, 100 μM and 1 mM were flowed through the setup. For convenience, we selected one of the quadrants to display the data, similar results were obtained for the remaining quadrants. Figure 5A shows the time dependence of the raw R_th_ data after sequential increase of the concentration of target molecules. No washing steps were performed during this measurement. In order to quantify the response better, the raw data are normalized here by dividing each R_th_ value (at a time t) by the initial baseline (time t = 0) in pure PBS and multiplying the outcome by 100 to get a normalized starting value of 100%. In short, the normalized value R_th_^norm^ (%) reads: $R_{\text{th}}^{\text{norm}}=\frac{R_{\text{th}}(t)}{R_{\text{th}(t=o)}}\times 100$. The error bars used in this manuscript are obtained by calculating the standard deviation on the average signal in each graph under equilibrium circumstances.

After the first addition (100 nM) an increase in R_th_ of 4.2% (±0.9%) was found. The maximum increase was at a concentration of 1 mM, resulting in a response of 24.0% (±1.3%) relative to the initial baseline level. Via this dose-response curve, the limit of detection (LOD) can be estimated. To determine this, the data is fitted with the function $R_{\text{th}}=X+Y\times \text{log}(\frac{C}{1\mu M})$, (*R*^2^ = 0.98) in the concentration regime of 0.1–100 μM. There is a linear relationship between the R_th_ and the log C for at least three orders of magnitude. The parameter of the fit are X = 92.40 and Y = 1.02. The limit of detection can be estimated to be lower than 100 nM, which is in the same order of magnitude as with the setup used in previous measurements without array features [30].

**Figure 6.**
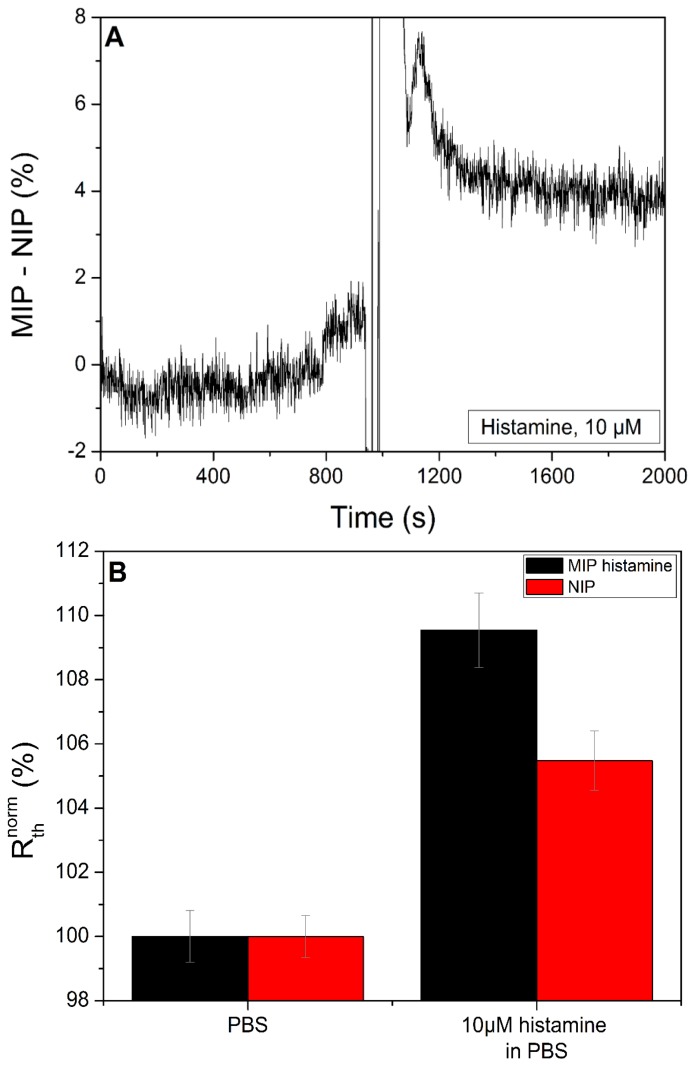
The graph in panel (**A**) shows a sample, onto which both a MIP and a NIP for histamine were applied, was allowed to stabilize with PBS (pH = 7.4). After stabilization the sample was exposed to a solution of histamine in PBS (pH = 7.4) with a concentration of 10 μM. Graph 6 (**B**) shows the average R_th_ as seen in Figure 6A plotted in function of the concentration.

**Figure 7.**
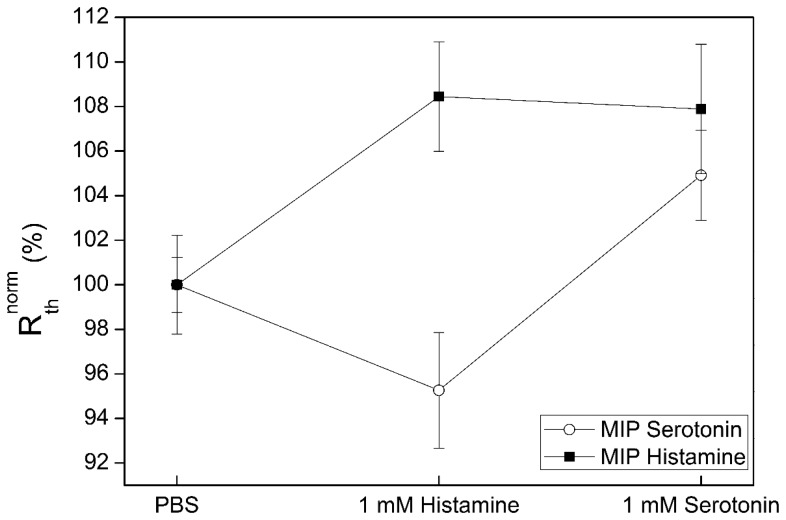
Thermal response of histamine- and serotonin-sensitive sensors spots upon exposure to a solution with a concentration of 1 mM histamine (first) and 1 mM serotonin (second) in PBS. The experiment has been performed by sequential additions of the target molecules to the sensor quadrants without intermittent washing steps.

The next step is determining the specificity of the sensor setup. For this purpose, two adjacent quadrants were coated with a histamine MIP and the other two were coated with the corresponding NIP, thus yielding a 50/50 ratio on the sample. Functionalizing a MIP and a NIP on a single sample gives the opportunity to perform differential measurements on the same chip, allowing the correction of non-specific binding within the same sample with a high level of confidence. The results are shown in Figure 6. After stabilization with PBS (pH = 7.4), a histamine concentration of 10 μM was introduced, resulting in an increase in both MIP and NIP sensor. To correct for the non-specific binding effect the normalized R_th_ of the NIP was subtracted from the R_th_ of the MIP. Previous research [15,17,23] has indicated that this operation facilitates the transition from well controlled buffer solutions to biological samples, such as urine, saliva or blood. Furthermore, external factors such as fluctuations of the environmental temperature, can be cancelled out. The result of this subtraction can be seen in Figure 7A. Figure 6B shows the average R_th_ value for each concentration of histamine and it can be seen that the MIP shows a bigger increase in R_th_ than the NIP for the highest concentration.

**Figure 8.**
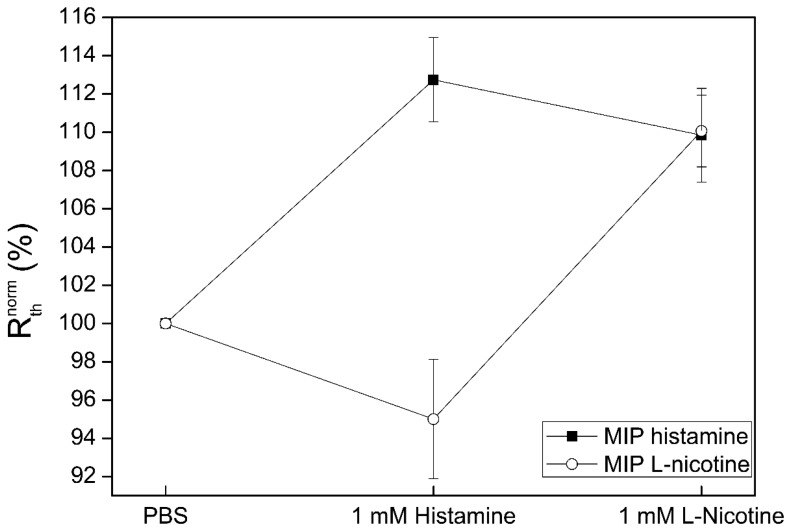
Thermal response curve of histamine and l-nicotine sensitive sensor spots upon exposure to a solution with a concentration of 1 mM histamine (first) and 1 mM l-nicotine (second) in PBS buffer.

To make a step towards an array format, the next experiment consisted of a sample onto which a MIP for histamine and a MIP for serotonin in a 50/50 ratio was applied. The sensor response is shown in Figure 7. After stabilization in PBS buffer, a concentration of 1 mM histamine was added to the setup. This concentration was chosen to be well in the saturation region to show the highest possible difference between the channels. As expected, the R_th_ of the histamine MIP sensor increases, indicating binding of the target molecule to the MIP layer. In turn, the R_th_ of the serotonin MIP experiences a slight decrease, most likely due to the presence of histamine in the fluid which may affect the overall thermal conductivity. The subsequent addition of a solution with a concentration of 1 mM serotonin resulted in a R_th_ increase for the serotonin MIP sensor, while there was no significant effect for the histamine MIP sensor. This shows that there is no significant cross selectivity between the serotonin and histamine MIP sensors despite the chemical similarity between the target molecules. The same measurement protocol, with a 50/50 ratio of surface coverage, was repeated for the MIP of histamine and the MIP of l-nicotine. The normalized data are shown in Figure 8.

After a stabilization period in PBS buffer, a concentration of 1 mM histamine was added to the setup. The R_th_ of the histamine MIP sensor increases as was expected, indicating binding of the target molecule to the MIP layer. Again, the R_th_ of the serotonin MIP experiences a slight decrease, which is thought to be due to the presence of histamine in the fluid which could affect the overall thermal conductivity. The subsequent addition of l-nicotine (1 mM) resulted in an increase in R_th_ for the l-nicotine MIP sensor. The histamine MIP sensor shows a slight decrease, in this case most likely caused by an altered thermal conductivity of the fluid due to the presence of l-nicotine. This indicates that there is no significant cross selectivity between the histamine and l-nicotine MIP sensors even though their size is similar.

## 4. Conclusions

In this work the heat transfer method for the detection of small organic molecules has been adapted to an array format using a new flow cell design based on the segmentation of the original version into quadrants. Relevant aspects regarding the performance of the novel setup have been tested, namely quadrant separation, limit of detection, specificity and cross selectivity.

Microscopy analysis demonstrated that the quadrants can be functionalized individually with MIP particles without interference, while maintaining a low level of intra-sample variability for sensor spots on the same chip. The dose response curve was determined on one individual quadrant with an l-nicotine MIP resulting in a limit of detection which is in the same order of magnitude as previous results.

In a next step, the quadrants were functionalized with 50/50 ratio of MIP and NIP in order to correct for non-specific binding. Upon exposure to its template molecule, the MIP-functionalized sensor displayed a stronger increase in R_th_ compared to the NIP sensor, showing the specificity of the system. To prove the array format capabilities of the novel sensor setup two experiments with MIPs for different targets were performed. The first was done with a sensor with a 50/50 area ratio of histamine- and serotonin MIPs, while the second consisted on an equivalent combination of histamine and nicotine. Even though the targets are chemically similar, the sensors demonstrated only a significant response to their corresponding target, indicating that there is no cross selectivity.

The novel heat-transfer method in combination with the miniaturized, array format flow cell makes it possible to measure the concentration of different target molecules up to the nanomolar range. Discrimination between similar small organic molecules was possible, showing no significant cross selectivity. Therefore, the MIP array sensor platform with HTM as a readout technique has the potential to become a low cost analysis tool for bioanalytical applications.
